# Development and evaluation of a peptide-based radiotracer for targeting TIM-3 in preclinical models

**DOI:** 10.3389/fimmu.2026.1744607

**Published:** 2026-07-23

**Authors:** Guangfa Bao, Jianfang Zhang, Yeqian Cui, Dan Li, Menglin Lu, Rencai Lu, Dongdong Xv, Mingkang Wu, Shaobo Wang

**Affiliations:** 1 Department of Nuclear Medicine, The First People’s Hospital of Yunnan Province, Kunming, Yunnan, China; 2 Yunnan Province Clinical Medical Center for Digestive System Diseases, Kunming, Yunnan, China

**Keywords:** immune checkpoint, peptide, PET/CT, T cell immunoglobulin mucin-3 (TIM-3), tracer

## Abstract

**Introduction:**

T cell immunoglobulin mucin-3 (TIM-3) plays a critical role in the T cell exhaustion and there have been already many related inhibitory antibodies in clinical trial. However, *in vivo* non-invasive visualization of TIM-3 is lagging in comparison to the development of therapeutic antibodies. This study aims to develop a positron emission tomography/computed tomography (PET/CT) imaging tracer targeting TIM-3 and explore its ability to visualize tumor lesions.

**Methods:**

A cysteine-modified, NOTA-conjugated peptide, NOTA-P24, was synthesized and radiolabeled with fluorine-18. Its stability and specificity were evaluated *in vivo* using uptake, competition, and efflux studies. Furthermore, PET/CT imaging and biodistribution studies were performed on tumor bearing animals.

**Results:**

[^18^F]AlF-NOTA-P24 showed high specificity and affinity to TIM-3-expressing cells *in vitro* and *in vivo*. Biodistribution studies on tumor-bearing mice demonstrated high tumoral uptake of the tracer and fast body clearance, resulting in high-contrast images and negligible exposure of healthy tissue to radiation.

**Conclusions:**

The work developed a peptide-based probe [^18^F]AlF-NOTA-P24 targeting TIM-3, with favorable specificity and affinity. This approach has the potential for noninvasive evaluation of TIM-3.

## Graphical Abstract

**Figure:**
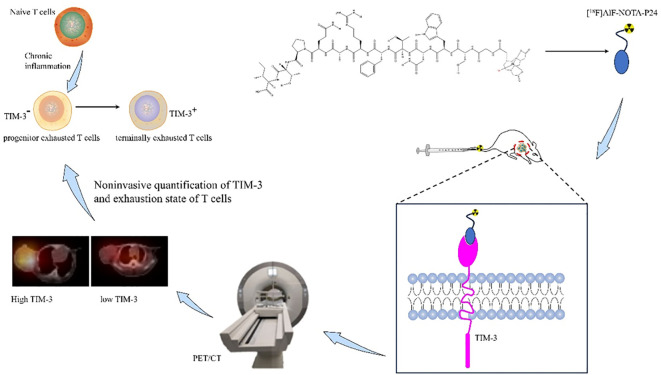

## Highlights

- Fluorine-18-labeled tracer targeting TIM-3 is lacking.
- The small-molecule peptide (P24) chosen for developing radiotracer has much higher affinity to TIM-3 than documented probe (^68^Ga-DOTA-P26) derived from P26.
- [^18^F]AlF-NOTA-P24 exhibited superior stability and specificity to TIM-3, excellent ability to visualize TIM-3 both *in vivo* and vitro.

## Introduction

Lung cancer remains the leading cause of cancer mortality worldwide and is categorized primarily as either small-cell lung cancer (SCLC) or non-small-cell lung cancer (NSCLC), with NSCLC representing approximately 85% of all cases (1, 2). Despite considerable advances in screening and personalized therapy in recent years, the five-year survival rate for lung cancer patients ranges only from 4% to 17%, depending on the disease stage and geographic region (2, 3). Immune checkpoint inhibitors (ICIs), exemplified by anti-programmed cell death 1 (PD-1) and anti-PD-ligand 1 (PD-L1) agents, now play a major role in treating advanced lung cancer (4). Nevertheless, only a subset of patients benefits from these costly therapies, and treatment responses are often accompanied by disease progression during or after the cessation of therapy (5, 6). Well-documented evidence indicates that T cell exhaustion is likely a principal underlying cause of these limitations (7–9).

The infiltration of cytotoxic CD8^+^ T cells into tumors strongly correlates with favorable outcomes in numerous cancers, including lung cancer (10). However, after continuous exposure to tumoral antigen, these T cells enter a differentiated state known as exhaustion, which is characterized by upregulated inhibitory receptors, diminished effector functions, reduced cytotoxicity, and metabolic alterations (9, 11, 12). Among these inhibitory molecules, T cell immunoglobulin mucin-3 (TIM-3) has emerged as a key immune checkpoint that suppresses immunity by impairing the maturation and activation of immune cells (10, 13, 14). Substantial evidence now indicates that TIM-3 is upregulated during resistance to immunotherapy, and its high expression on T cells may contribute to adaptive resistance against anti-PD-1 or anti-CTLA-4 treatments (15, 16). Consequently, TIM-3 blockade could act synergistically with other immune checkpoint inhibitors to enhance antitumor immunity (14, 16–18). Several phase 1/2 clinical trials evaluating anti-TIM-3 antibodies are currently underway, predominantly in combination with anti-PD-1/L1 monoclonal antibodies, and have shown activity in NSCLC patients previously progressing on anti-PD-1 therapy (19, 20).

With growing interest in TIM-3 blockade therapy, non-invasive methods to identify patients most likely to respond are urgently required. Positron emission tomography/computed tomography (PET/CT), a key molecular imaging technique, provides a favorable platform for the non-invasive assessment of TIM-3 expression. Currently, only a few TIM-3-targeting tracers exist, such as ^64^Cu-NOTA-RMT3-23, ^124^I-C23, ^125^I-C23 and ^68^Ga-DOTA-P26 ^(^21–23). However, the first three are antibodies with high molecular weight and long circulating time, requiring several days between injection and imaging. Although the peptide P26 has higher bioactivity in blocking TIM-3, its affinity is substantially lower than that of P24 (affinity constant (KD): 3.44 × 10^-5^ M vs. 9.50 × 10^-8^ M) (patent No.CN111533785A) (24). The superior affinity of P24 makes it a more suitable candidate for imaging applications. Consequently, we developed a modified P24 peptide and constructed a fluorine-18-labeled TIM-3 targeting tracer, [^18^F]AlF-NOTA-P24, then evaluated its pharmacokinetics and specific diagnostic potential in preclinical models.

## Methods

### Bioinformatic analysis

Clinical and molecular data for 523 patients with lung adenocarcinoma (LUAD), including gender, age, grade, histological type, TNM categories, pathologic stages, and survival outcomes, were obtained from the TCGA database (https://portal.gdc.cancer.gov) and served as the training cohort. Subsequent analyses, including survival and immune infiltration assessments, were performed using R software version 4.5.1 (https://www.r-project.org/).

### Construction of the precursor and fluorescent peptide

P24 (SEQ: GSWNTFRAQPTI) is a novel peptide which specifically targets TIM-3 verified by earlier documented research and patent (24). We conjugated the macrocyclic chelator 1, 4, 7-triazacyclononane-N, N’, N″-triacetic acid (NOTA) to this peptide via an amide reaction for radiolabeling. A fluorescent analogue was also prepared by conjugating fluorescein isothiocyanate (FITC) to yield FITC-Acp-P24, which incorporates an alkyl spacer between the terminal amino group and the thiourea linkage. Purification by semi-preparative HPLC yielded a final purity exceeding 95%.

### Molecular docking

The molecular structure of NOTA conjugated precursor (termed as NOTA-P24) was constructed in ChemDraw and Chem3D. The human TIM-3 protein structure (PDB ID: 5F71) was obtained from the RCSB Protein Data Bank (https://www.rcsb.org/). PyMol software (https://pymol.org/) was then used to remove redundant protein chains and ligand molecules. Following dehydration, hydrogenation, and energy minimization of both the TIM-3 protein and NOTA-P24, molecular docking was performed using PyRx software (https://pyrx.sourceforge.io/) equipped with Autodock and Autodock Vina. The docking grid box was set to encompass the entire protein structures. The model exhibiting the lowest binding energy was selected as the optimal binding configuration. PyMol was subsequently used to visualize the docking results.

### Cell line

Human non-small cells lung cancer cells (A549) were cultured in complete DMEM medium supplemented with 10% fetal bovine serum and 100U/mL penicillin-streptomycin at 37 °C in a humidified atmosphere with 5% CO_2_. Highly TIM-3-expressing A549^TIM-3^ cells were transfected by lentivirus encompassing human TIM-3 plasmid (Obio Technology, Inc., China) and selected using 3 μg/mL puromycin (Beyotime, China).

### Immunofluorescence and western blot

Immunofluorescence was performed to verify whether transfection is successful and to assess the TIM-3 expression on A549^TIM-3^ cells. Briefly, A549^TIM-3^ cells were seeded in 6-well plates overnight, fixed with methanol for 15 minutes, permeated with 0.5% Triton X-100, blocked with 5% bovine serum albumin (BSA) for 60 minutes. Then, the cells were incubating primary antibodies at 4 °C overnight. After washing out the primary antibodies (Clone no.4C4G3, Proteintech, China), fluorescein-conjugated secondary antibodies (Cat no. RGAR004, Proteintech, China) were added and cultured in the dark for 60 minutes at room temperature. The cells were stained with DAPI, examined using an inverted fluorescence microscope. The A549 cells were used for negative control.

Similarly, A549 and A549^TIM-3^ cells were lysed with RIPA buffer. Protein concentration was determined using a BCA assay kit (P0010S, Beyotime, China). Equal amounts of protein were loaded into each lane, separated by electrophoresis, and transferred to a PVDF membrane. The membrane was blocked with 5% milk and then incubated overnight with primary antibodies against TIM-3 and actin (Clone no.2D4H5, Proteintech, China). Following incubation with a horseradish peroxidase-conjugated secondary antibody for one hour at room temperature, the membrane was visualized using an enhanced chemiluminescence assay (MA0186, Meilunbio^®^, China).

### Radiochemistry and stability

The synthesis of precursor, NOTA-conjugated P24 peptide, was manufactured by Hefei Taikubio Co., Ltd. The radiolabeling of [^18^F]AlF-NOTA-P24 was referred to the research described previously (25). Briefly, [^18^F]fluoride was trapped on an activated Sep-Pak light QMA cartridge (WAT023525, Waters, USA), which was sequentially activated by 5 mL normal saline and 10 mL double distilled water (ddH_2_O), and then trapped [^18^F]fluoride was eluted with 0.2 mL of a 2% saline/acetonitrile solution (50/50, v/v). A solution containing 50 nmol of the NOTA-P24 precursor dissolved in 200 μL of 0.2 M acetate buffer (pH = 4) was then combined with 50 nmol of AlCl_3_ (5 nmol/μL, dissolved by 0.2M pH=4 acetate buffer) in a vial, and thereafter 200 μL saline/acetonitrile elution containing 37GBq ^18^F was added into the vial. The mixture was stirred with a magnetic stir bar and reacted at 110 °C for 15 min, during which a C18 Sep-Pak Light solid-phase extraction cartridge (WAT023501, Waters, USA) was activated by 5 mL absolute ethyl alcohol and 10 mL ddH_2_O. The reaction mixture was cooled down and purified using the activated C18 cartridge. the C18 cartridge was then rinsed by 30 mL ddH_2_O for removing un-conjugated ^18^F^-^, the product adsorbed to the cartridge was eluted by 500 μL ethanol/ddH_2_O solution (50/50, v/v) and passed the 0.22 μm filter for sterilization. At the end of purification, non-decay-corrected radiolabeling yields were measured and recorded.

Stability of [^18^F]AlF-NOTA-P24 in phosphate buffer saline (PBS) and FBS were monitored from 1 h to 4 h using high performance liquid chromatography equipped with a symmetry C18 Column (WAT054275, Waters, USA) and a radioactivity detector (radio-HPLC). [^18^F]AlF-NOTA-P24 was incubated with PBS and FBS for 1, 2 and 4 hours at 37°C. To remove proteins from FBS, an equal volume of acetonitrile (MeCN) was added to denature and precipitate the proteins. The mixture was then centrifuged at 3000 rpm for 6 minutes to remove the precipitated serum proteins. The supernatant was subsequently collected for sample analysis. Mobile phase A consisted of linear gradient elution using 0.1% TFA/MeCN and 0.1% TFA/H_2_O, maintaining 0% MeCN for 5 minutes for complete absorption to column, starting from 10% MeCN at the 5th minutes and a 3% increase per minute at 1 mL/min flow rate.

### LogP measurement

A mixture containing 5 KBq of [^18^F]AlF-NOTA- P24 was added to a system comprising 0.5 mL of n-butyl alcohol and 0.5 mL of water (pH = 7.4). The mixture was vigorously vortexed for 15 minutes and then centrifuged at 12, 500 rpm for 5 minutes to ensure complete phase separation. Subsequently, 4×100 µL aliquots were collected from each of the organic and aqueous phases. The radioactivity of each aliquot was measured using a gamma counter, and the counts per minute (CPM) were recorded. The Log P value was calculated based on these measurements. The log P value was calculated using the following formula: log P = log [(mean CPM in n-butyl alcohol)/(mean CPM in water)].

### *In vitro* characterization of [^18^F]AlF-NOTA-P24

*In vitro* binding studies were conducted using A549 and the stably transfected human TIM-3 cell line A549^TIM-3^. Competition experiments were performed by simultaneous exposure to unlabeled precursor (10^−11^ to 10^−7^ M) and radiolabeled compound for 60 min in A549^TIM-3^ cells. For the cell uptake assay, A549 and A549^TIM-3^ cells (2×10^6^) were seeded in 6-well plates overnight and the next day adherent cells were incubated with 74 KBq [^18^F]AlF-NOTA-P24, after 0.5 h, 1h and 2 h incubation at 37 °C, cells were washed and quantified in a gamma counter (Wizard 2480, Perkin Elmer Instruments Inc, USA). Blockade experiment was performed by adding excess unlabeled NOTA-P24 (100 nmol) together with 74 KBq [^18^F]AlF-NOTA-P24. Internalization assay was performed like to the uptake assay, the membrane-bound fraction was separated by incubating cells for 10 min in 1 mL 50 mM glycine and 100 mM NaCl, pH2.8, then internalized fraction was collected by lysing cells in 1 mL 0.1 M NaOH.

### Animal models

Female BALB/c nu/nu mice (age = 6-8weeks) were housed under standard laboratory conditions. 1× 10^7^ A549 or A549^TIM-3^ cells in serum-free media were injected at the right shoulder. Tumor volume was closely monitored using a vernier caliper and calculated using the formula: tumor volume = (length × width²)/2. When tumors reached a volume of 500 mm^3^, all mice were injected with tracers and performed PET/CT scans. Given the limitation of our PET/CT instrument, which is designed for human imaging, A549^TIM-3^ bearing models were also established on female nude rats (100-120g) for dynamic PET/CT imaging. Sample sizes were selected based on standard practice in the field (26).

### IVIS imaging

Three A549^TIM-3^ tumor-bearing nude mice were selected and fasted overnight prior to the experiment to minimize interference from food autofluorescence on the experimental results. FITC-Acp-P24 was then (100 μL, 1 mM)administered via tail vein injection, and imaging was performed using the IVIS Spectrum *in vivo* optical imaging system. Before imaging, the nude mice were placed in a small animal anesthesia machine and induced with 5% isoflurane. The tumor-bearing mice were then positioned appropriately within the IVIS Spectrum and maintained under anesthesia with 1% isoflurane. Fluorescence signals were acquired and imaged at an excitation wavelength of 495 nm and an emission wavelength of 519 nm to evaluate the *in vivo* targeting capability of the fluorescent peptide.

### Biodistribution

Biodistribution studies were conducted using mice with xenografted A549^TIM-3^ tumors. Mice were injected with 3.7 MBq [^18^F]AlF-NOTA-P24 and sacrificed at 0.5, 1, 2h and 4h respectively (n = 5). The mice were euthanized using carbon dioxide anesthesia. Briefly, after placement in the euthanasia chamber, carbon dioxide (CO_2_) was introduced at a uniform flow rate displacing 30%-70% of the chamber volume per minute to rapidly induce unconsciousness and minimize distress. The animals lost consciousness within 2–3 minutes. Respiratory activity and eye color were continuously monitored for each animal. After all animals had ceased breathing, CO_2_ administration was maintained for at least one additional minute. Then, tumors and organs were quickly collected, weighed and counted for radioactivity by a γ-counter. The background- and decay-corrected counts per minute (CPM) for each sample were represented as percent of injected dose per gram of tissue (% ID/g). Of note, %ID/g is used for biodistribution because it gives the absolute, ex vivo measure of radioactivity per gram of tissue, another index-the maximal standardized uptake values (SUVmax) is used for *in vivo* imaging because it provides a non-invasive, weight-normalized index of tracer uptake that can be tracked longitudinally.

### PET/CT imaging

For imaging, the radiotracer in PBS solution (150 μL, pH = 7.2) was injected into the tail vein of mice. At 1, 2, 3 and 4 h after injection, images were obtained using PET/CT (Philips Ingenuity TF PET/CT scanner). The mice were anesthetized using 5% isoflurane and keep anesthetic using 1% isoflurane (inhaled), and static PET scans were acquired over 10 min. A scout scan followed by a CT scan was routinely acquired prior to PET imaging. The CT parameters were: collimation width 9.6 mm, pitch 0.9375, rotation time 0.8 s, and tube settings of 80 kV and 160 mAs. All PET/CT images were reconstructed into DICOM (Digital Imaging and Communications in Medicine) format. PET reconstruction employed the Ordered Subsets Expectation Maximization (OSEM) algorithm (4 iterations) with scatter, attenuation, and decay corrections. Regions of interest (ROIs), including the liver, muscle, tumor, and kidney, were delineated on CT images owing to the relatively low tissue resolution of PET. The corresponding PET/CT fusion images were referenced simultaneously to guide this delineation while avoiding areas susceptible to spillover Effect. SUVmax was then calculated for each ROI. For blocking research, the static PET/CT images were first obtained as aforementioned. Then the next day, the same mice were simultaneously injected with cold NOTA-P24 (10 mg/kg) and [^18^F]AlF-NOTA-P24, images were obtained at 1 h after injection.

For dynamic PET/CT imaging, rats were anesthetized with isoflurane (5% for induction, 1% for maintenance) and fitted with indwelling needles. CT images were acquired prior to radiopharmaceutical injection. Following placement in the PET scanner, each rat received a rapid injection of 11.1 MBq [^18^F]AlF-NOTA-P24, and the PET acquisition commenced simultaneously. The dynamic scan was divided into 20 frames of 180 seconds each. SUVmax for major organs-including tumors, liver, kidneys, and muscle-were recorded to generate time-activity curves (TACs).

### Immunohistochemistry

IHC procedures and scoring for TIM-3 staining were carried out for evaluate the expression levels of TIM-3. Briefly, the tumor tissues of the mice were fixed in 4% paraformaldehyde, followed by embedding in paraffin. Then the tissues were sliced into 4-μm sections, and then re-hydrated, retrieved, blocked, and stained with appropriate primary (Cat No.11872-1-AP, Proteintech, China) and secondary antibodies (GB23303, Servicebio, China). Positive staining was detected via a 3, 3’-diaminobenzidine (DAB) reaction. The sections were then counterstained with hematoxylin and imaged on a bright-field microscope.

### Biosafety evaluation

To evaluate the probe’s safety, healthy female BALB/c-nu/nu mice (16-18 g, 6-8 weeks old) were randomly assigned to acute toxicity (24 h post-injection) or chronic toxicity (7 d post-injection) groups and administered 37 MBq of [^18^F]AlF-NOTA-P24. Blood samples were collected for routine hematology and biochemistry analyses. The heart, liver, spleen, lung, and kidney were harvested for histopathological examination.

### Statistics

All quantitative data are expressed as the mean ± standard deviation. Differences between two or more groups were analyzed using Student’s t test or one-way ANOVA with consecutive LSD-t tests in SPSS 27.0 software. Differences of *P* < 0.05 indicate statistical significance.

## Results

### Bioinformation

Survival analysis of the TIM-3 gene (HAVCR2) within the cohort revealed that elevated HAVCR2 expression correlated with a higher patient risk score and increased mortality (**Supplementary Figure 1A**). Furthermore, HAVCR2 expression was positively associated with the stromal score, immune score, and the total score (estimate score), suggesting a link to improved efficacy of immune checkpoint blockade in PD1-positive and CTLA-4/PD1 dual-positive patients (**Supplementary Figures 1B, C**). HAVCR2 expression also correlated with the overexpression of immunosuppressive genes, including LAIR1, PDCD1LG2, CTLA-4, and PDCD1 (**Supplementary Figure 2A**), and with increased infiltration of suppressive immune cells such as M2 macrophages, neutrophils, and activated CD4^+^ memory T cells (**Supplementary Figure 2B**). Collectively, these findings indicate that HAVCR2 likely plays an unfavorable role in LUAD.

### Molecular docking

**Figure 1:**
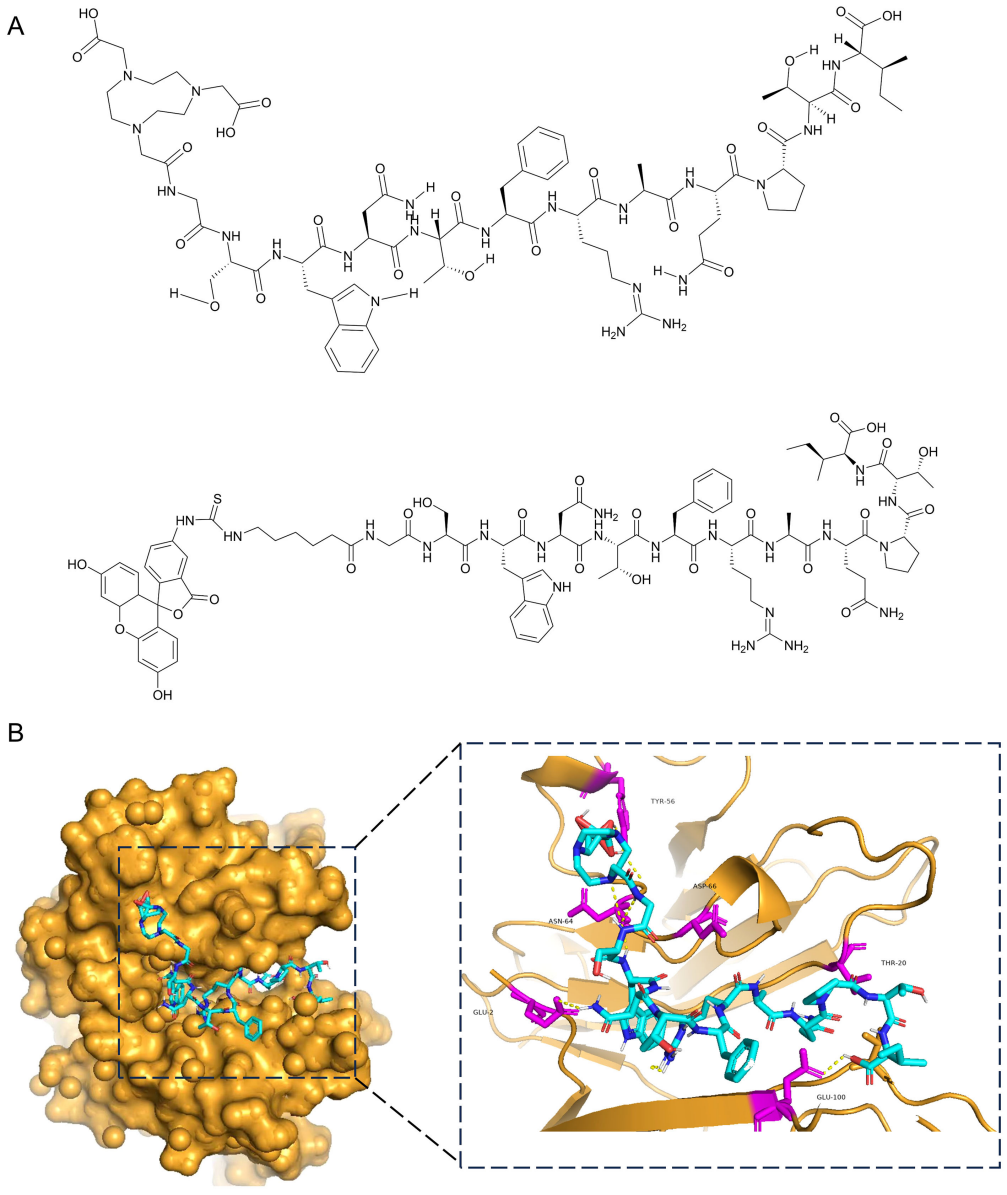
**(A)** Overview of structure of NOTA-P24 (NOTA-GSWNTFRAQPTI) and fluorescent peptide (FITC-Acp-GSWNTFRAQPTI). **(B)** Molecular docking analysis of NOTA-P24 with human TIM-3 protein (ID: 5F71).

NOTA-P24 (**Figure 1A**) was synthesized and characterized using mass spectrometry, with a calculated m/z value of 1663.05 for [M + 2 H]2+ ion of C_74_H_111_N_21_O_23_; the experimental value was found to be 1663.05 (**Supplementary Figure 3A**). Similarly, a fluorescein 5-isothiocyanate (FITC)-conjugated form, FITC-Acp-P24 (**Figure 1A**; **Supplementary Figure 3B**), was produced and characterized. The binding affinity between NOTA-P24 and human TIM-3 protein was determined via molecular docking by PyRx software and exhibited the interactive interface using PyMol software. As illustrated in **Figure 1B**, NOTA-P24 exhibited strong binding to TIM-3, with ligand-mediated recognition involving residues Glu2, Thr20, Tyr56, Asn64, Asp66 and Glu100, and an optimized binding energy of -8.2 kcal/mol.

### Immunofluorescence and western blot

**Figure 2:**
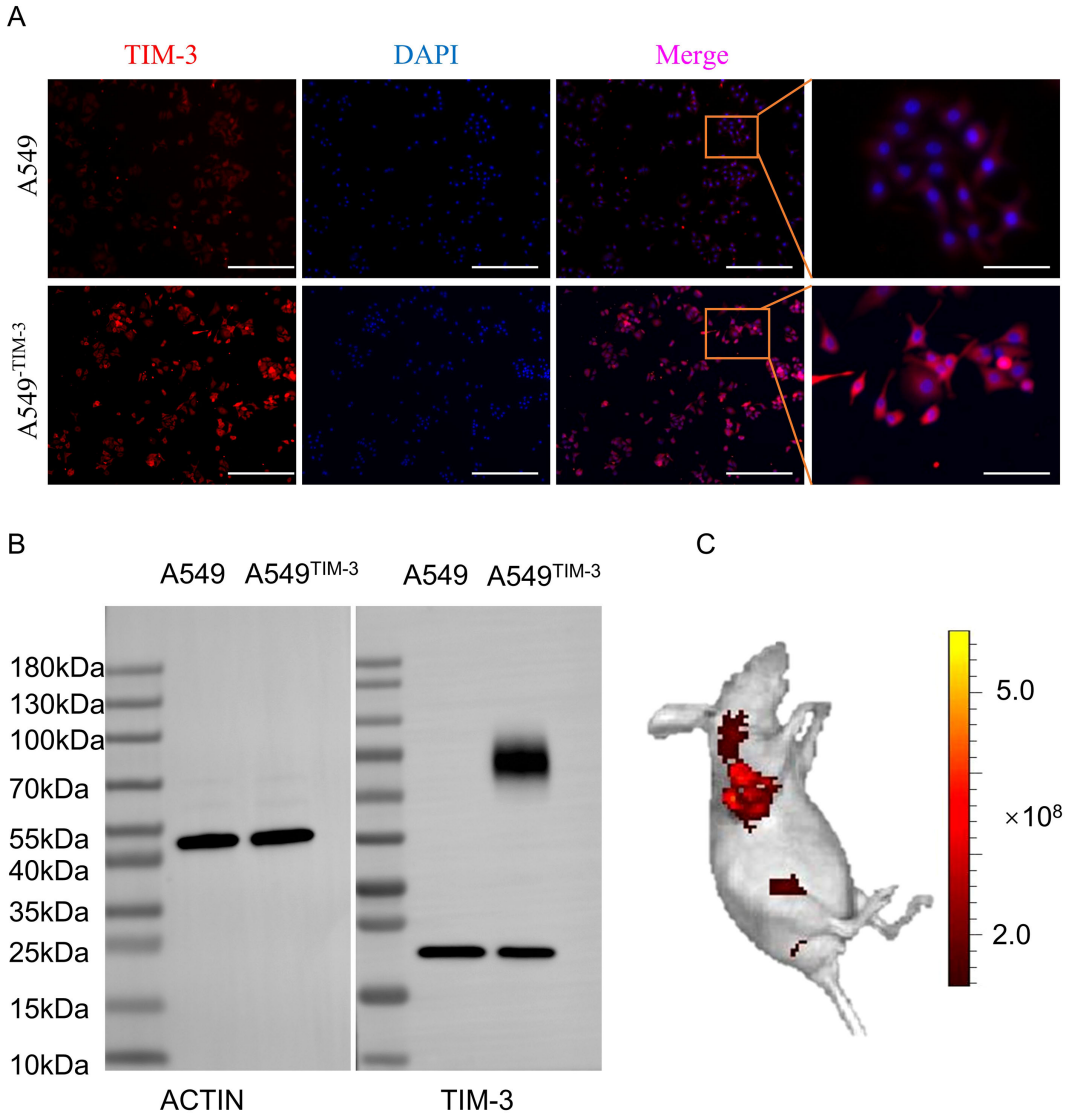
The verification of cellular trans-infection and tumoral model. **(A)** Immunofluorescence imaging of A549 and A549^TIM-3^, immunofluorescent TIM-3 (red) and DAPI (blue) staining of a field of A549 and A549^TIM-3^, Scale bar = 25 μm. **(B)** TIM-3 immunoblotting of A549 and A549^TIM-3^. **(C)** IVIS imaging of FITC-Acp-P24 in mice bearing A549^TIM-3^ tumor (n = 3).

Immunofluorescence and western blot analyses evaluated TIM-3 expression in A549^TIM-3^ cells after trans-infection. Immunofluorescence imaging revealed a substantial increase in TIM-3 levels on these cells compared to the wild-type control (**Figure 2A**). Western blot analysis similarly confirmed the marked overexpression of TIM-3 in A549^TIM-3^ cells (**Figure 2B**). Interestingly, the A549^TIM-3^ sample exhibited two distinct bands, likely due to post-translational modification. Furthermore, IVIS imaging of mice bearing A549^TIM-3^ tumors detected a clear green fluorescence signal (**Figure 2C**). Subsequent IHC staining (**Supplementary Figure 4**) verified pronounced TIM-3 overexpression in the tumor-bearing mice, confirming the successful establishment of the A549^TIM-3^ cell line and demonstrating the suitability of this mouse model for further investigation.

### Radiochemistry

The radiosynthesis was performed manually and completed within 40 minutes, affording a non-decay-corrected radiochemical yield of 15.3 ± 6.1% (n = 10) in the absence of acetonitrile. This yield increased significantly to 30.5 ± 8.3% (n = 10) when acetonitrile was included. The improvement is likely due to the tracer’s hydrophilic character, as indicated by its measured log P value of 0.78. The radiochemical purity of [^18^F]AlF-NOTA-P24 exceeded 90% after purification, as determined by HPLC analysis (**Supplementary Figures 5A, B**). the molar activity was about 29.8 MBq/nmol (29.8 ± 5.4, n=10). The tracer’s retention time was approximately 7.5 min. To evaluate its stability, [^18^F]AlF-NOTA-P24 was incubated in PBS and FBS for 1, 2, and 4 hours, followed by radio-HPLC quantification. The proportion of intact tracer remained above 90%, demonstrating favorable stability (**Supplementary Figure 5C**).

### *In vitro* characterization of [^18^F]AlF-NOTA-P24

**Figure 3:**
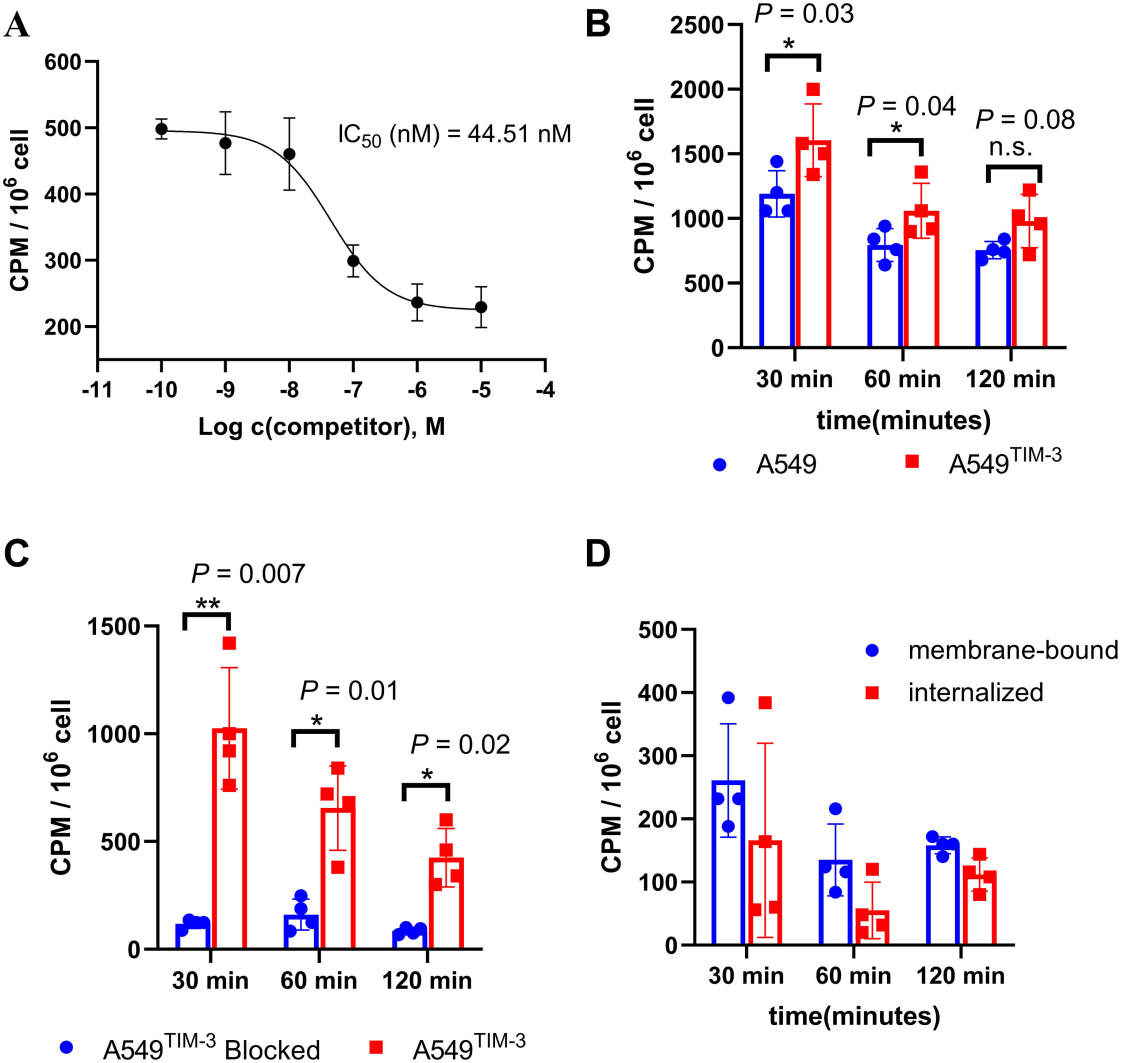
*In vitro* binding specificity, cellular uptake, blockade experiment and internalization analysis of [^18^F]AlF-NOTA-P24. **(A)** Competition experiments. A549^TIM-3^ cells were simultaneously exposed to unlabeled precursor (10^-11^ to 10^-7^ M) and [^18^F]AlF-NOTA-P24 for 60 min. **(B)** Cellular uptake of [^18^F]AlF-NOTA-P24 in A549 control and A549^TIM-3^ cells. **(C)** Cellular blockade experiments. Excess unlabeled NOTA-P24 (100 nmol) was added into A549^TIM-3^ cells together with [^18^F]AlF-NOTA-P24. **(D)** Analysis for membrane bound and internalized fraction of [^18^F]AlF-NOTA-P24. Error bars represent the SD. * *p* < 0.05, **p < 0.01, ***p < 0.001, ****p < 0.0001.

[^18^F]AlF-NOTA-P24 specifically bound to human TIM-3-expressing cells with a half-maximal inhibitory concentration of 44.51 nM, which was comparable to the affinity of P24 for human TIM-3 (95 nM) (**Figure 3A**) (24). Cellular uptake differed substantially between A549 and A549^TIM-3^ cells (1190.00 ± 179.26 vs 1605.00 ± 281.60 CPM/10^6^ cells, *P* = 0.03 at 30 min; 795 ± 126.89 vs 1060 ± 212.29 CPM/10^6^ cells, *P* = 0.04 at 60 min)(**Figure 3B**). Blocking assays also revealed a significant reduction in cellular radioactivity within the blocked group compared to controls (1025.00 ± 281.60 vs 118 ± 20.78 CPM/10^6^ cells, *P* = 0.007 at 30 min; 655.00 ± 195.53 vs 161.00 ± 72.10 CPM/10^6^ cells, *P* = 0.01 at 60 min; 425.00 ± 135.03 vs 85.00 ± 15.45 CPM/10^6^ cells, *P* = 0.02 at 120 min; **Figure 3C**), further confirming the specificity of this tracer. Cell-based internalization assays showed rapid uptake of [^18^F]AlF-NOTA-P24 into the cells, with 38.87% of the total bound fraction located intracellularly after 30 minutes of incubation. Over 4 hours, only a marginal decrease in activity occurred, and 41.48% of the bound tracer remained internalized (**Figure 3D**).

### Biodistribution

**Figure 4:**
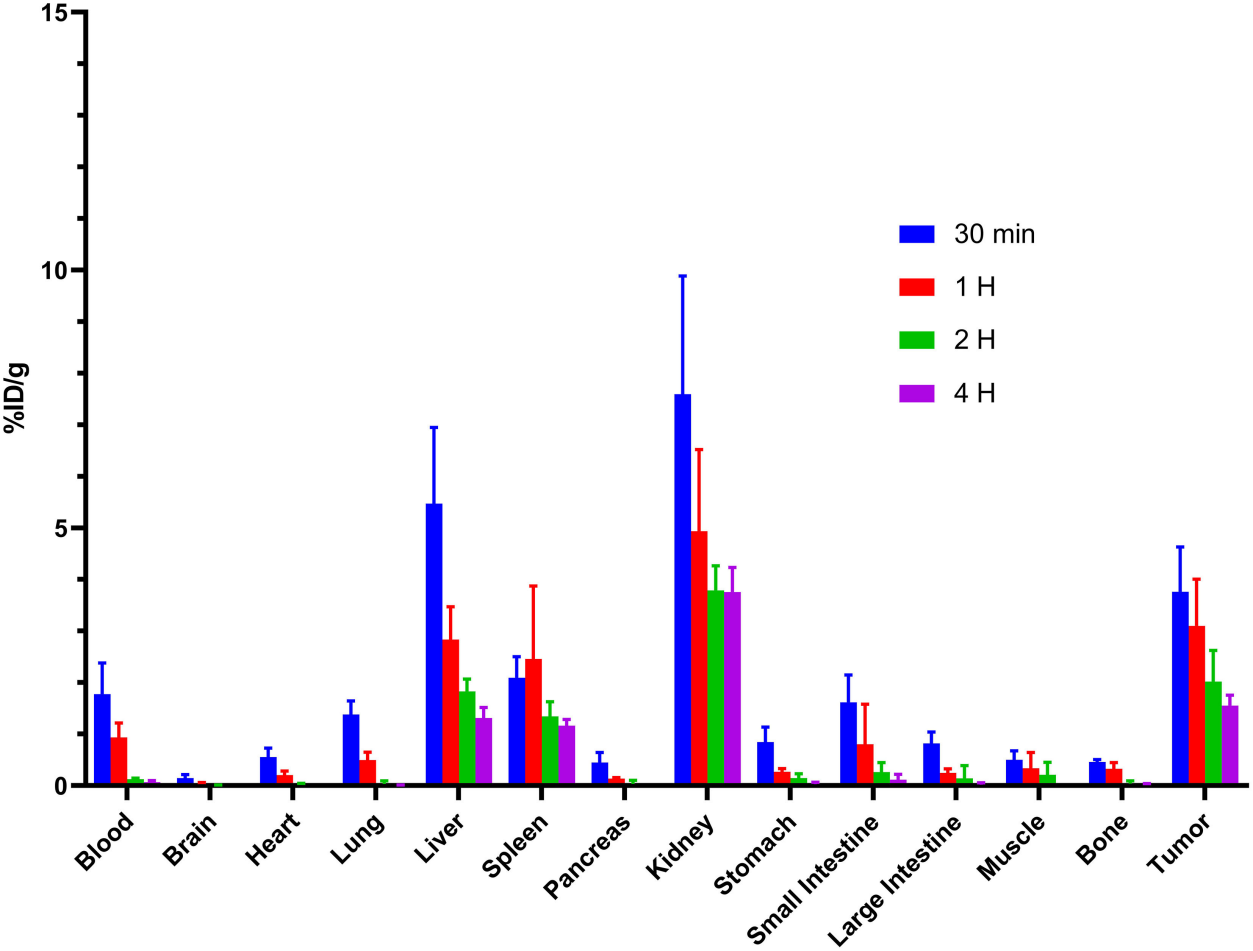
Biodistribution of [^18^F]AlF-NOTA-P24 in A549^TIM-3^ xenografts at 30 min, 1 h, 2 h and 4 h after radiotracer administration (n = 5 respectively).

The biodistribution of [^18^F]AlF-NOTA-P24 was investigated in A549^TIM-3^ cells-bearing mice (n=5; **Figure 4**; **Supplemental Table 2**). The radioactivity in kidneys was predominant with an uptake value of 7.59 ± 2.29%ID/g at 30 min post-injection, and 4.94 ± 1.58%ID/g at 60 min, indicating rapid urinary excretion. Among normal tissues, the spleen exhibited notably high accumulation of [^18^F]AlF-NOTA-P24, reaching 2.46 ± 1.41%ID/g at 60 min. Tumors also demonstrated substantial tracer uptake, peaking at 3.76 ± 0.87%ID/g 30 min after injection. The tumor-to-muscle ratios of the probe were approximately 7.5, 9.2, and 9.6 at 0.5, 1, and 2 h (The CPM was set to 0 mentioned as follows due to measurement difficulties at 4h), respectively. Most normal organs showed low uptake, particularly the brain, heart, lung, pancreas, and muscle, which even fell below the detection limit of the γ counter by 4 h post-injection. Activity in part organs including brain, heart, lung, pancreas, digestive tract, and muscle had fallen below the detection limit of the γ counter, for the purpose of calculation, the CPM value of this organ region was set to 0 (**Supplemental Table 1**).

### PET/CT imaging

**Figure 5:**
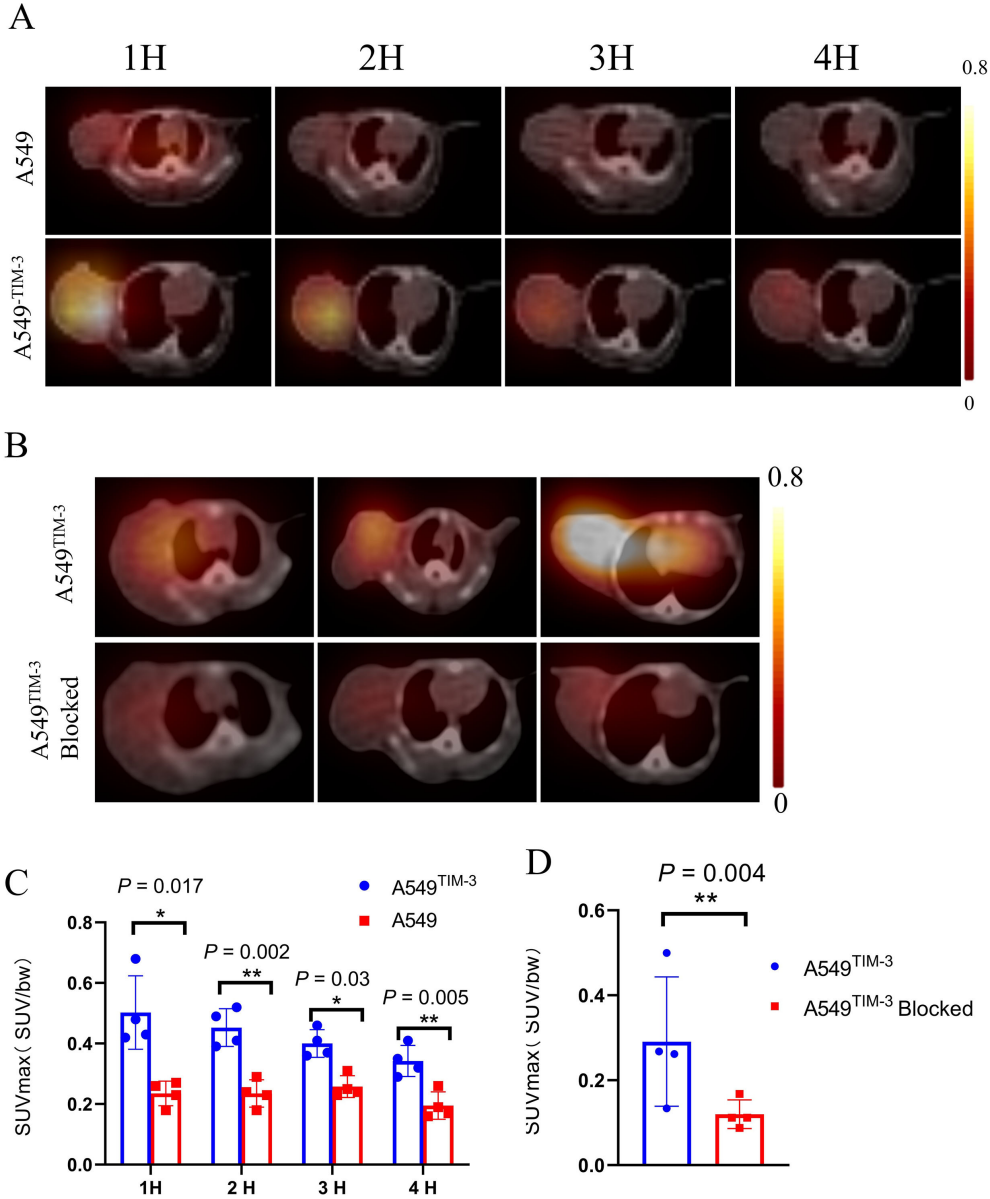
**(A)** PET imaging of [^18^F]AlF-NOTA-P24 in mice bearing A549 or A549^TIM-3^ tumors (n = 4). **(B)** PET imaging in mice bearing A549^TIM-3^ tumors with and without pre-injection of unlabeled NOTA-P24 as competitor (n = 3). **(C)** Quantification of tumoral SUVmax in mice bearing A549 or A549^TIM-3^ tumors. **(D)**Contrast for tumoral SUVmax between mice with and without pre-injection of unlabeled NOTA-P24. Error bars represent the SD. * *p* < 0.05, **p < 0.01, ***p < 0.001, ****p < 0.0001.

The low uptake and rapid elimination in non-target organs resulted in high imaging contrast. Tumor lesions showed substantial [^18^F]AlF-NOTA-P24 uptake and favorable tumor-to-background ratios (**Figures 5A**, **C**; **Supplemental Table 2**). Pre-injection of NOTA-P24 significantly reduced [^18^F]AlF-NOTA-P24 accumulation (**Figures 5B**, **D**; **Supplementary Figure 6**). The tumor-to-muscle ratio for A549^TIM-3^ tumors reached 10.3 at 60 min, compared to 1.7 for A549 tumors and 2.6 for the blocking group. Together, these findings confirmed the specificity of [^18^F]AlF-NOTA-P24.

**Figure 6:**
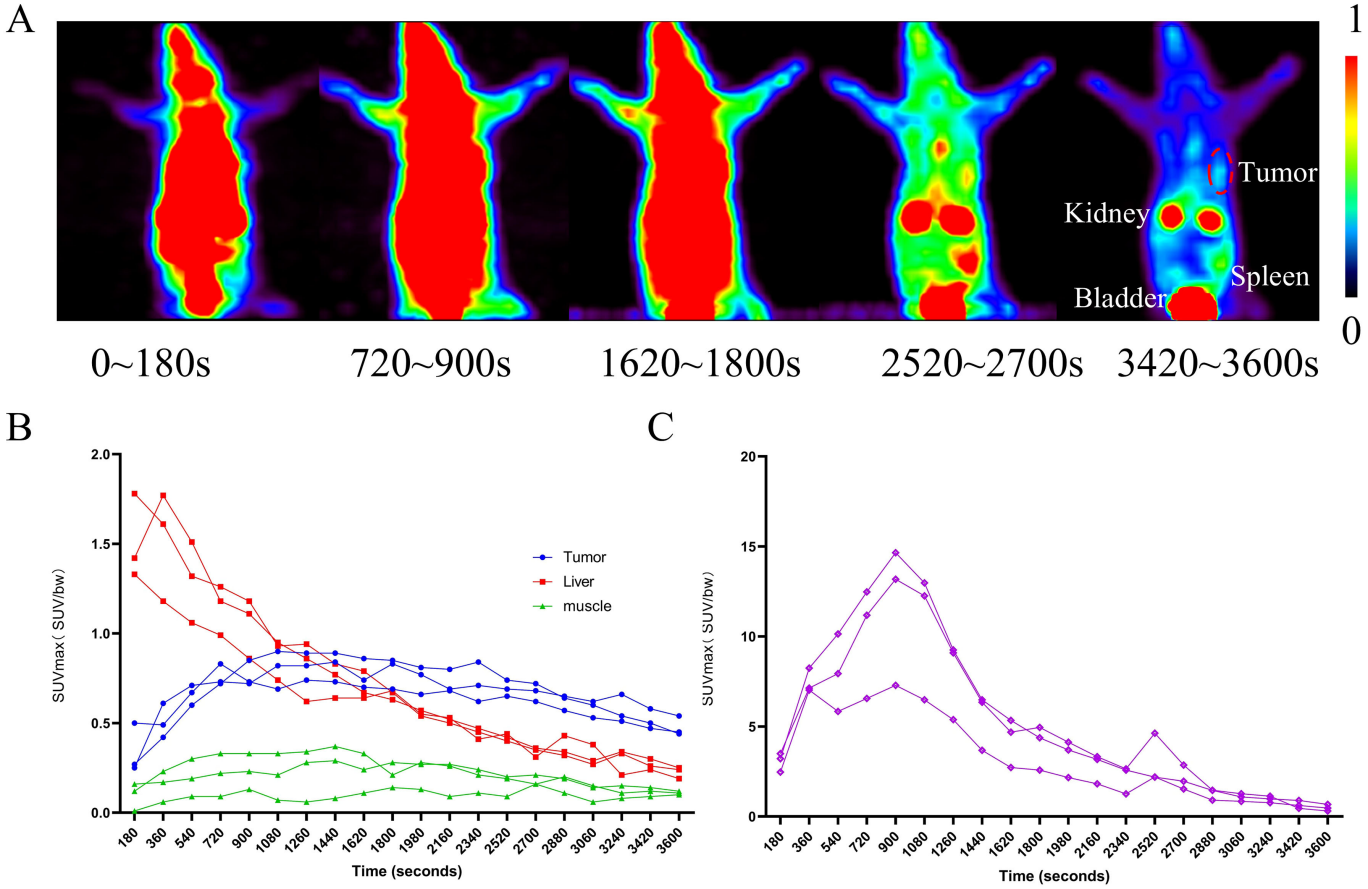
Representative dynamic [^18^F]AlF-NOTA-P24 PET/CT images in A549^TIM-3^ bearing nude rats in different time **(A)** and time-activity curves (TAC) for tumors, livers, muscles **(B)** and kidneys **(C)** (n = 3).

Dynamic whole-body images of rats were displayed as maximum intensity projections (MIPs). As shown in **Figures 6A–C**, renal excretion served as the primary clearance route for [^18^F]AlF-NOTA-P24, aligning with biodistribution data. Muscle and bone exhibited minimal probe accumulation, corresponding to low background radioactivity and high-contrast images. Excellent tumor-to-background images were obtained as early as 45 minutes post-injection. The spleen, as an immune organ, displayed significant [^18^F]AlF-NOTA-P24 accumulation, consistent with biodistribution results (**Figure 6A**; **Supplementary Figure 7**). This evidence indicates that the radiolabeled tracer effectively visualizes TIM-3 expression levels.

### Toxicity

**Figure 7:**
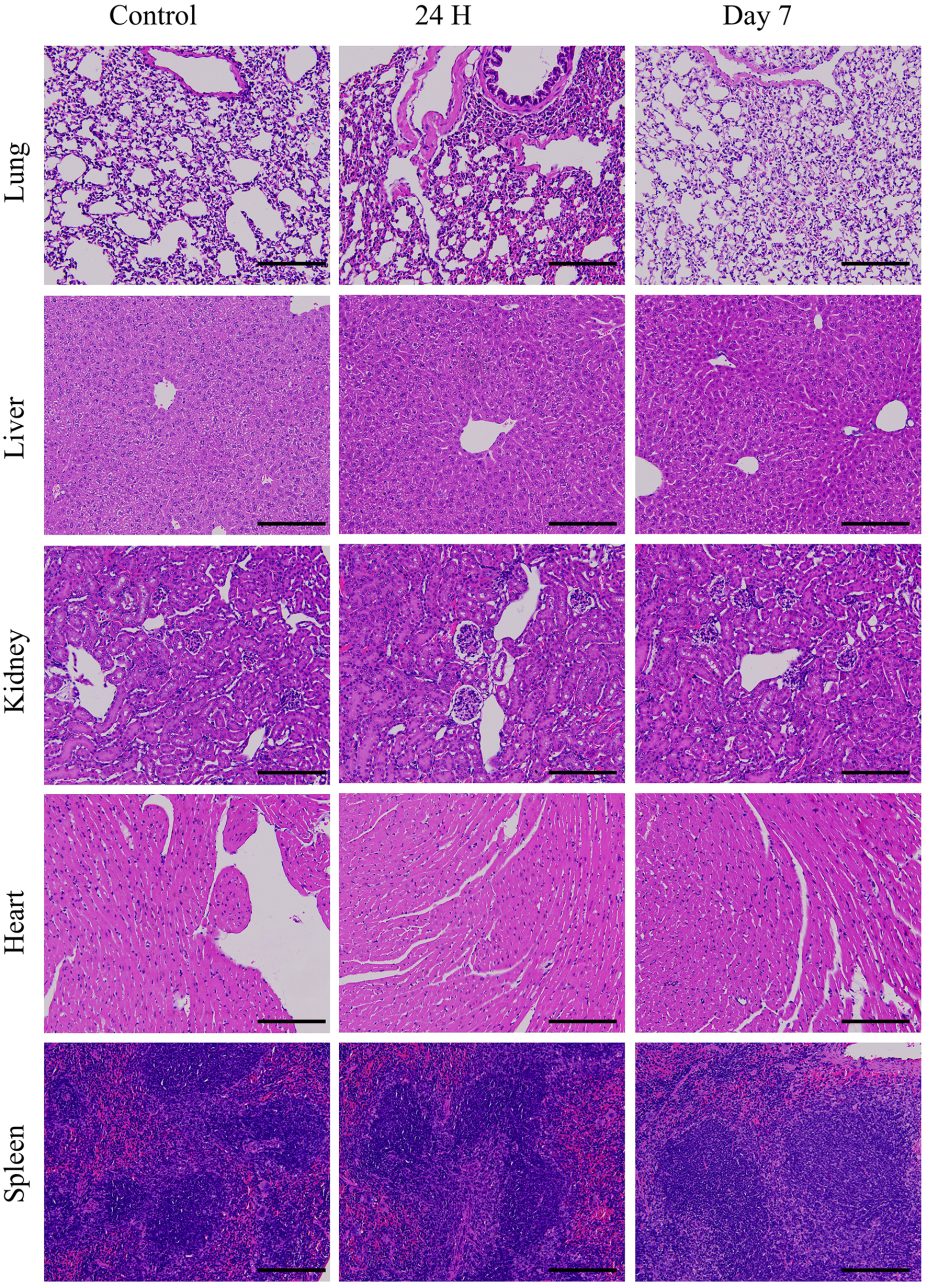
Representative images of hematoxylin & eosin staining (×200, Bar = 50 μm). Mice suffered from administration of saline and 37 MBq [^18^F]AlF-NOTA-P24 were sacrificed after 24 h and 7 days, significant organs including heart, lung, liver, kidney and spleen were collected for H&E staining (n =3).

No adverse events occurred, and no significant changes in vital signs or laboratory parameters were observed. In mice injected with [^18^F]AlF-NOTA-P24, the counts of WBCs, lymphocytes, monocytes, and granulocytes all remained within normal ranges, indicating no hematological toxicity (**Supplemental Table 3**). Serum biochemical analysis confirmed the absence of kidney or liver damage (**Supplemental Table 4**). Furthermore, hematoxylin and eosin staining showed no anatomical abnormalities in the heart, lung, liver, spleen, or kidney (**Figure 7**).

## Discussion

To our knowledge, no tracers radiolabeled with ^18^F nuclide have been reported for specifically targeting TIM-3, we therefore conducted the first *in vivo* investigation of the [^18^F]AlF-NOTA-P24 imaging agent, which is based on a series of functional TIM-3 binding peptides. The lead candidate peptide P24 was conjugated with a NOTA chelator, radiolabeled with ^18^F, and its properties were evaluated *in vitro* and *in vivo*. [^18^F]AlF-NOTA-P24 exhibited favorable characteristics for an ideal tracer, including good apparent affinity, satisfactory radiochemical purity, excellent stability, high tumor uptake, and favorable tumor-to-background ratios shortly after administration.

Immune checkpoint blockade (ICB) has emerged as a major therapeutic approach in oncology by reinvigorating exhausted T cells (27). However, the efficacy of these drugs is often limited, potentially due to the presence of other immune checkpoint molecules such as TIM-3 (28). Recent clinical trials demonstrate that dual blockade of TIM-3 and PD1/PD-L1 produces synergistic antitumor effects. In a phase I/II clinical trial (NCT02608268), combined anti-TIM-3 (sabatolimab) and anti-PD1 (spartalizumab) therapy provided preliminary evidence of antitumor efficacy with favorable tolerability (18, 19). Identifying patients who may benefit from TIM-3-targeted therapies is therefore crucial. The noninvasive nature of PET/CT imaging, along with its repeatability, high sensitivity, accuracy, and specificity, makes it well-suited for monitoring TIM-3 *in vivo*. Visualizing TIM-3 expression in tumors enables better assessment of exhausted T cell status and supports the development of personalized treatment regimens.

The peptide-based tracer [^18^F]AlF-NOTA-P24 presents several advantages relative to antibody-based tracers, such as its smaller size for enhanced tumor penetration and rapid renal clearance, along with more straightforward engineering modification and chemical synthesis. Furthermore, the precursor is less costly to obtain. The radionuclide ^18^F is also generally more cost-effective and easier to produce than alternatives like ^68^Ga, ^64^Cu and ^89^Zr, which improves its clinical accessibility.

The favorable pharmacokinetics, high affinity, and metabolic stability of [^18^F]AlF-NOTA-P24 support its suitability for the noninvasive visualization of TIM-3. Conjugation to NOTA did not compromise the affinity of peptide P24, yielding an IC50 of 44.51 nM, which is comparable to that of P24 alone (95 nM) (**Figure 3A**) (24). To our knowledge, ^68^Ga-DOTA-P26 is the only peptide-based tracer derived from the same patent as ours, yet according to the patent manuscript, P26 exhibits far superior bioactivity to P24 in blocking TIM-3, even though its affinity is markedly lower than that of P24 (affinity constant [KD]: 3.44 × 10^-5^ M vs. 9.50 × 10^-8^ M). An optimal binding affinity not only ensures the specific interaction of the probe with its target but also facilitates its rapid clearance from non-target organs, thereby achieving the higher possible target-to-background ratio. Additionally, three probes were developed from antibodies (^64^Cu-NOTA-RMT3-23, ^124^I-C23, ^125^I-C23), which exhibit high affinity but require a waiting period of several days after injection before imaging (21–23). From this perspective, P24 might be the more suitable one for developing as a radiotracer.

The radiotracer was produced with high radiochemical purity and molar activity (**Supplementary Figure 5**). Competition experiments (**Figure 3A**), cellular blockade assays (**Figure 3C**) *in vitro*, and markedly reduced accumulation in A549 tumors along with blocked A549^TIM-3^ tumors (**Figure 5**) further confirmed its specificity. Although its log P value of 0.78 indicates moderate lipophilicity, [^18^F]AlF-NOTA-P24 accumulation was primarily observed in the kidneys in both biodistribution studies and PET/CT imaging, the tracer also exhibited high splenic uptake in rats. The elevated signal in the spleen largely reflects the abundance of TIM-3-positive immune cells, including myeloid and NK cells, pointing to target-specific binding. This implies that [^18^F]AlF-NOTA-P24 may be capable of assessing TIM-3 expression not only in tumor cells but also in immune cells. The probe is predominantly cleared through the kidneys, as evidenced by the swift accumulation of radioactivity in the renal cortex and pelvis, followed by its passage into the bladder via urine. This clearance pattern is typical of low-molecular-weight peptides (<5 kDa) undergoing glomerular filtration. Such a metabolic profile helps reduce background interference from the hepatobiliary and gastrointestinal tracts (**Figures 4**–**6**). However, when interpreting imaging findings of abdominal lesions, careful attention should be paid to the physiologically high radiotracer uptake in the spleen and kidneys. Finally, [^18^F]AlF-NOTA-P24 caused almost no detrimental effects in major organs (**Figure 7**; **Supplemental Table 3**, **4**), suggesting a favorable safety profile for further clinical translation.

Although our study demonstrates the potential of [^18^F]AlF-NOTA-P24 for monitoring TIM-3 expression *in vivo*, several limitations remain. First, [^18^F]AlF-NOTA-P24 is derived from a linear peptide, which offers advantages such as ease of synthesis, low immunogenicity, and rapid tissue penetration. However, it may also exhibit lower *in vivo* stability than cyclic peptides, despite favorable stability was observed in FBS (**Supplementary Figure 5**). Our future work will focus on improving its stability through strategies such as incorporating non-natural amino acids, terminal capping, and PEGylation, while maintaining an appropriate balance between binding affinity and metabolic properties. Furthermore, since our investigation focused primarily on lung cancer, the applicability of [^18^F]AlF-NOTA-P24 to other malignancies remains unclear. Future studies will evaluate its diagnostic performance across diverse tumor types. Thirdly, because our PET/CT system is designed for human imaging, its resolution is insufficient for distinguishing murine organs, resulting in poor image quality in mice. Therefore, dynamic PET/CT imaging was conducted in nude rats bearing A549^TIM-3^ tumors. Fourth, improving the compound’s hydrophilicity represents another important objective for further development. Last but no least, because our institution lacks the qualification to conduct human or patient experiments, [^18^F]AlF-NOTA-P24 has not been translated to the clinic here for testing tumors with natural TIM-3 expression.

## Conclusion

On the basis of a lead peptide, [^18^F]AlF-NOTA-P24 was developed to quantify TIM-3 expression in the tumor microenvironment using PET/CT. Our results demonstrated superior properties of [^18^F]AlF-NOTA-P24 including high affinity, satisfactory radiochemical purity, excellent stability, pronounced tumor uptake, and favorable tumor-to-background ratios shortly after administration. Together, these findings underscore the radiotracer’s potential for noninvasive assessment of TIM-3 expression and T-cell exhaustion state.

## Data Availability

The datasets generated during and/or analyzed during the current study are available from the corresponding author on reasonable request.
