# Supplementary material for: Development and evaluation of a peptide-based radiotracer for targeting TIM-3 in preclinical models
Source: Front Immunol. 2026 Jul 23;17:1744607. doi: 10.3389/fimmu.2026.1744607 (PMC13442668; doi:10.3389/fimmu.2026.1744607)
Supplement: Supplementary file 1 [file SupplementaryFile1.docx]

**Title page**

**Development and evaluation of a peptide-based radiotracer for targeting TIM-3 in preclinical models**

Guangfa Bao^1,2#^, Jianfang Zhang^1#^, Yeqian Cui^1^, Dan Li^1^, Menglin Lu^1^, Rencai Lu^1^, Dongdong Xv^1^, Mingkang Wu^1^, Shaobo Wang^1,2*^

Affiliations:

^1^Department of Nuclear Medicine, The First People's Hospital of Yunnan Province, Kunming, 650032, Yunnan, China.

^2^Yunnan Province Clinical Medical Center for Digestive System Diseases, Kunming, 650032, Yunnan, China.

Corresponding Author:

*Prof. Shaobo Wang, Department of Nuclear Medicine, The First People's Hospital of Yunnan Province, Kunming, 650032, Yunnan, China. Tel: +86-15812082912, Email: wshbo_98@126.com

Supplemental Fig.1 Bioinformatic analysis of TIM-3 gene (termed as HAVCR2) in lung adenocarcinoma (LUAD) patients. (A) the relationship between HAVCR2 gene and patients’ prognosis. (B)the difference of stromal score, immuneScore and the total score (ESTIMATEScore) between patient spectrum with or without high expression of HAVCR2. (C) and (D) The correlation of HAVCR2 expression level and therapeutic efficacy of immune checkpoint blockade for CTLA^-^PD1^+^ (C) and CTLA^+^PD1^+^ (D) patients.

Supplemental Fig.2 (A) the correlation among HAVCR2 gene with other genes. (B) the relationship between HAVCR2 gene expression level with immune cells.

Supplemental Fig.3 The mass spectrometry report of NOTA-P24 (A) and FITC-Acp-P24 (B).

Supplemental Fig.4 H&E and Immunohistochemistry staining of A549 tumors and A549^TIM-3^ tumors (n=4). Scale bar = 50 μm.

Supplemental Fig.5 Quality control and in vitro stability analysis of [^18^F]AlF-NOTA-P24. (A) In vitro characterization of NOTA-P24 and [^18^F]AlF-NOTA-P24 via HPLC. (B) the stability evaluation of [^18^F]AlF-NOTA-P24 after incubating with PBS and FBS in 37℃ for 1 h, 2 h and 4 h.

Supplemental Fig.6 (A)Representative MIP images of PET imaging of [^18^F]AlF-NOTA-P24 in mice bearing A549 or A549^TIM-3^ tumors (n = 4). (B) MIP imaging in mice bearing A549^TIM-3^ tumors with and without pre-injection of unlabeled NOTA-P24 as competitor (n = 3).

Supplemental Fig.7 Representative dynamic [^18^F]AlF-NOTA-P24 PET/CT images in A549^TIM-3^ bearing nude rats (n = 3).

Supplemental Fig.8 acute and chronic toxicity evaluation in functions of liver and kidney via blood biochemistry (ALT, AST, BUN and CREA). The two dashed lines represent the reference range for normal values.

Tables

Supplemental Table 1. The results for [^18^F]AlF-NOTA-P24 biodistribution (n = 5)

| tissue/organ | 30 min | 60 min | 120 min | 240 min |
| --- | --- | --- | --- | --- |
| Blood | 1.78±0.60 | 0.93±0.28 | 0.12±0.02 | 0.07±0.03 |
| Brain | 0.14±0.07 | 0.03±0.02 | 0.02±0.01 | NA |
| Heart | 0.55±0.18 | 0.20±0.08 | 0.03±0.01 | NA |
| Lung | 1.38±0.26 | 0.49±0.15 | 0.04±0.03 | NA |
| Liver | 5.47±1.48 | 2.83±0.64 | 1.83±0.24 | 1.31±0.21 |
| Spleen | 2.09±0.41 | 2.46±1.41 | 1.34±0.29 | 1.16±0.12 |
| Pancreas | 0.45±0.20 | 0.13±0.02 | 0.03±0.02 | NA |
| Kidney | 7.59±2.29 | 4.94±1.58 | 3.79±0.47 | 3.75±0.48 |
| Stomach | 0.84±0.29 | 0.27±0.06 | 0.14±0.08 | 0.03±0.02 |
| Small Intestine | 1.62±0.53 | 0.80±0.71 | 0.26±0.19 | 0.11±0.07 |
| Large Intestine | 0.82±0.22 | 0.24±0.08 | 0.14±0.11 | 0.02±0.01 |
| Muscle | 0.50±0.18 | 0.34±0.21 | 0.21±0.18 | NA |
| Bone | 0.46±0.05 | 0.33±0.12 | 0.04±0.02 | 0.01±0.03 |
| Tumor | 3.76±0.87 | 3.10±0.90 | 2.02±0.60 | 1.55±0.20 |

NA: not available (cannot be detectable)

Supplemental Table 2. The contrast of SUVmax between A549 and A549^TIM-3^ tumors (n = 4)

| Time | A549^TIM-3^ | A549 | *P* |
| --- | --- | --- | --- |
| 1H | 0.50±0.12 | 0.24±0.04 | * *P*=0.017 |
| 2 H | 0.45±0.06 | 0.25±0.05 | * **P*=0.002 |
| 3 H | 0.40±0.05 | 0.26±0.04 | * *P*=0.03 |
| 4 H | 0.34±0.05 | 0.20±0.05 | ** *P*=0.005 |

Supplemental Table 3. The results of blood routine test (n = 3)

| Parameters | 24 H | 7 Day | Units | Reference Ranges |
| --- | --- | --- | --- | --- |
| WBC | 2.33±1.05 | 4.01±0.89 | 10^9/L | 0.80 - 10.60 |
| Neu | 0.39±0.18 | 0.9±0.51 | 10^9/L | 0.23 - 3.60 |
| Lymph | 1.65±0.81 | 2.62±0.62 | 10^9/L | 0.60 - 8.90 |
| Mon | 0.16±0.08 | 0.29±0.09 | 10^9/L | 0.04 - 1.40 |
| Neu% | 16.40±0.56 | 24.26±7.50 | % | 6.5 - 50.0 |
| Lym% | 69.95±3.46 | 60.68±28.39 | % | 40.0 - 92.0 |
| Mon% | 6.60±0.42 | 7.81±0.88 | % | 0.9 - 18.0 |
| RBC | 7.46±0.67 | 9.33±1.88 | 10^12/L | 6.50 - 11.50 |
| HGB | 117.5±9.19 | 105.87±34.58 | g/L | 110 - 165 |
| HCT | 42.45±3.32 | 44.83±10.45 | % | 35.0 - 55.0 |
| MCV | 46.90±0.71 | 47.01±7.44 | fL | 41.0 - 55.0 |
| MCH | 15.70±0.14 | 16.54±1.62 | pg | 13.0 - 18.0 |
| MCHC | 276.00±12.78 | 311.32±90.88 | g/L | 300 - 360 |
| RDW-CV | 16.60±1.41 | 17.15±6.07 | % | 12.0 - 19.0 |
| RDW-SD | 30.20±4.10 | 31.24±7.04 | fL | 23.0 - 39.0 |
| PLT | 1238.50±252.43 | 1348.66±159.20 | 10^9/L | 400 - 1600 |
| MPV | 5.40±0.28 | 5.91±1.57 | fL | 4.0 - 6.2 |
| PDW | 16.50±0.28 | 15.1±3.68 |  | 12.0 - 17.5 |
| PCT | 0.504±0.21 | 0.42±0.27 | % | 0.100 - 0.780 |

Supplemental Table 4. The results of biochemical analyses (n = 3)

| Parameters | 24 H | 7 Day | Units | Reference Ranges |
| --- | --- | --- | --- | --- |
| ALT | 55.87±32.98 | 64.09±10.63 | U/L | 10.06-96.47 |
| AST | 189.84±46.29 | 161.47±54.29 | U/L | 36.31-235.48 |
| BUN | 17.86±2.14 | 18.24±3.25 | mg/dL | 10.81-34.74 |
| CREA | 50.39±5.52 | 24.03±1.53 | μmol/L | 10.91-85.09 |
